# Cluster-randomised trial of the impact of an obesity prevention intervention on childcare centre nutrition and physical activity environment over 2 years

**DOI:** 10.1017/S1368980021004109

**Published:** 2022-11

**Authors:** Ruby Natale, Folefac D Atem, Cynthia Lebron, M Sunil Mathew, Sitara M Weerakoon, Catherina Chang Martinez, Karla P Shelnutt, Rachel Spector, Sarah E Messiah

**Affiliations:** 1 Department of Pediatrics, University of Miami Miller, School of Medicine, Miami, FL 33130, USA; 2 University of Texas Health Science, Center School of Public Health, Dallas Campus, Dallas, TX, USA; 3 Center for Pediatric Population Health, Children’s Health System of Texas, UTHealth School of Public Health, Dallas, TX, USA; 4 Department of Public Health Sciences, University of Miami Miller, School of Medicine, Miami, FL, USA; 5 Nova Southeastern University, Davie, FL, USA; 6 Department of Family, Youth and Community Sciences, University of Florida, Gainesville, FL, USA; 7 The Children’s Trust, Miami, FL, USA

**Keywords:** Obesity prevention, Early childhood, Childcare centre

## Abstract

**Objective:**

The prevalence of obesity among pre-school-aged children in the USA remains unacceptably high. Here, we examine the impact of Healthy Caregivers-Healthy Children (HC2) Phase 2, a childcare centre (CCC)-based obesity prevention intervention on changes in the CCC nutrition and physical activity environment over 2 school years.

**Design:**

This was a cluster-randomised trial with twelve CCC receiving the HC2 intervention arm and twelve in the control arm. The primary outcome was change in the Environment and Policy Assessment and Observation (EPAO) tool over 2 school years (Fall 2015, Spring 2016 and Spring 2017). Changes in EPAO physical activity and nutrition score were analysed via a: (1) random effects mixed models and (2) mixed models to determine the effect of HC2 *v.* control.

**Setting:**

The study was conducted in twenty-four CCC serving low-income, ethnically diverse families in Miami-Dade County.

**Participants:**

Intervention CCC received (1) teachers/parents/children curriculum, (2) snack, beverage, physical activity, and screen time policies, and (3) menu modifications.

**Results:**

Two-year EPAO nutrition score changes in intervention CCC were almost twice that of control CCC. The EPAO physical activity environment scores only slightly improved in intervention CCC *v.* control CCC. Intervention CCC showed higher combined EPAO physical activity and nutrition scores compared to control CCC over the 2-year study period (*β* = 0·09, *P* = 0·05).

**Conclusions:**

Obesity prevention programmes can have a positive impact on the CCC nutrition environment and can promote healthy weight in early childhood. CCC may need consistent support to improve the physical activity environment to ensure the policies remain intact.

US childhood obesity rates remain unacceptably high with apparent disparities by pre-school age; 16·5 % of Hispanics, 11·6 % of non-Hispanic Blacks, 9·9 % of non-Hispanic White and 7·0 % of Asians have obesity at the age of 2 to 5 years^(1)^. Moreover, higher obesity prevalence estimates among ethnic minority groups are often underscored by low socio-economic status, limited access to affordable fresh fruits and vegetables, and safe spaces to be physically active^(2)^. Adding more challenge, our team found that most parents with a pre-school-aged child with an unhealthy weight from low-income backgrounds thought their child was normal weight^(3)^.

The prevalence of obesity in pre-schoolers is noteworthy as several studies have shown that early paediatric onset of obesity tracks into adulthood^(4–7)^. In fact, overweight or obese pre-school children are at least five times more likely than non-obese children to be overweight or obese as adults^(4)^. The pre-school years in particular are a critical period in which long-term dietary and physical activity behaviours are established and have long-reaching effects on health^(8)^. The nutritional environment of a young child is complex and influenced by energy intake, intake of sweetened beverages, fruit and vegetable consumption, and caregiver feeding practices^(9)^. Similarly, the physical activity environment of a pre-school-aged child depends on factors like time allocated to active play, screen time and time spent outside^(10,11)^.

Studies have shown that obesogenic behaviours such as lack of physical activity, poor dietary outcomes and screen time in pre-school-aged children can be impacted by interventions in the childcare setting^(12,13)^. Multi-component, multilevel early care and education interventions with parental engagement have been reported to be particularly effective^(13,14)^. Childcare settings are a potential influential infrastructure to implement early childhood health interventions because: (1) 70 % of pre-school-aged children are enrolled in daily, out-of-home childcare; (2) low-income children consume much of their RDA in the childcare setting and (3) many children spend the majority of their waking hours in this setting^(15)^. As such, the Institute of Medicine’s Standing Committee on Childhood Obesity Prevention created a set of policy recommendations designed to prevent obesity in early childhood by promoting healthy early environments in settings outside the home where young children spend substantial time and identified childcare settings in particular^(16)^. Indeed, family, childcare and community environment factors that affect food intake and physical activity emphasise the role of the environment in relationship to health are all important to establishing healthy habits early in life^(17,18)^.

As such, here we report the results of ‘Healthy Caregivers – Healthy Children’ (HC2) over 2 school years, a childcare centre (CCC)-based obesity prevention programme couched in the socioecological model. The socioecological model model recognises that for prevention strategies to be successful, they must be implemented on multiple levels that include person, social and community networks and factors, as well as existing policies^(8–10)^. This randomised controlled trial specifically addresses the role of the CCC as change agents by implementing best-practice policies that are critical to intervention maintenance and an effective mediator of change. Our primary outcome of interest was change (e.g. improvement) in the CCC nutrition and physical activity environment to ultimately support healthy weight during these critical years of development. It was hypothesised that those CCC randomised to HC2 would show improvement in the nutrition and physical activity environment to support healthy weight among child attendees *v.* control centres. A secondary aim was to perform a separate analysis to explore a joint model that included the combined EPAO nutrition and physical activity subscores as one outcome.

## Methods

The present analyses used data from a randomised controlled intervention trial (Clinical Trial # NCT01722032) of HC2. Details of the study design can be found elsewhere^(18)^. We briefly summarise the design and intervention below. The university Institutional Review Board approved this study protocol, and each child’s parent or legal guardian provided informed consent to participate in the study.

### Study design

**Fig. 1:**
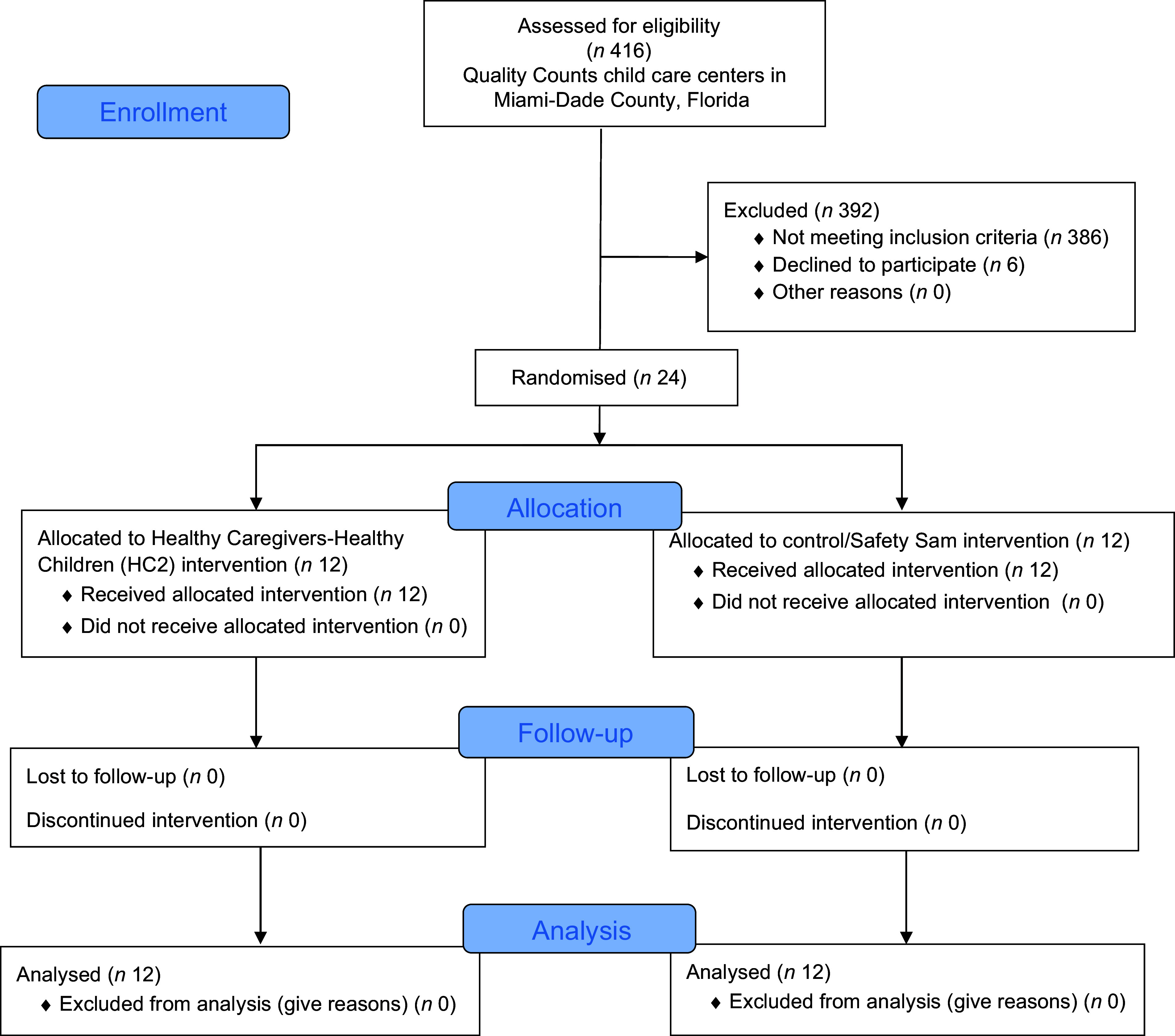
CONSORT 2010 flow diagram

This randomised controlled trial was conducted among twenty-four CCC serving low-income, ethnically diverse families in Miami-Dade County (MDC), FL, from August 2015 to June 2017. Random assignment of individuals to treatments was not feasible due to the CCC setting (no ability to randomise individuals within classrooms to treatments); rather we randomly assigned, via a random numbers generator programme, CCC to one of two treatments; thus, this study was designed as a group-randomised trial, also called a *cluster*-randomised trial^(19)^. CCC were randomly assigned (via the RAND or ‘uniform’ call in SAS) to one of two arms: (1) control arm that received an attention control (*n* 12 centres); or (2) intervention arm that received HC2 (*n* 12 centres). Both arms were followed and/or received treatment for 2 school years (approximately 9 months each), and outcome measures described below were collected at three key time points: the beginning of school year 1 (Fall 2015) and the end of school year 1 and 2 (Spring 2016 and 2017) (see Fig. 1, CONSORT flow diagram). In the first phase of HC2, the intervention was conducted by the research team and resulted in positive changes in dietary intake and BMI growth trajectories among those randomised to the treatment arm^(20)^. In this second phase of HC2, a train-the-trainer approach was implemented using the HC2 obesity prevention toolkit on parent and teacher adoption of healthy lifestyle role modelling behaviours and policy integration and compared to an attention control group^(18)^.

### Participants

This study was conducted in collaboration with MDC’s Quality Rating Improvement Centers ‘Quality Counts’ CCC and the University of Florida Institute of Food and Agricultural Sciences (UF/IFAS) Extension Family Nutrition Program (FNP) staff from MDC^(18)^. Specifically, Quality Counts is a voluntary rating system that reviews independent (e.g. not Head Start or affiliated with the local school system) CCC (not homes) according to clearly defined, high-quality standards using support and incentives to help providers reach their goals. To be included in the trial, Quality Counts CCC must have met the following criteria: (1) > 50 children aged 2 to 5 years old enrolled (but could also enrol infants to age 2); (2) serve low-income families and (3) mirror the ethnic composition of the MDC Public School System (63 % Hispanic, 19 % non-Hispanic Black and 18 % non-Hispanic White). CCC were excluded if they had a high prevalence of children with intellectual and developmental disabilities. Children with food allergies and sensitivities were included if their parent consented to study participation. Children who brought their own meals due to diet restrictions and those who were identified by parents on the demographic form as failure to thrive (< 5th BMI or BMI %ile) were excluded. CCC directors and teachers consented to participate at the beginning of study, and parents and their children were invited to participate.

### Healthy caregivers, healthy children (HC2) intervention

#### Detailed description of HC2 toolkit

The HC2 toolkit consists of material designed to incorporate all current nutrition and physical activity policy requirements for Florida pre-school children and embrace best-practice guidelines from Caring for Our Children^(21)^, Healthy Kids, Healthy Future (formerly Let’s Move!^(22)^), and USDA Team Nutrition^(23)^.

#### Tier 1: environmental changes, policy component

The following policies serve as the foundation of HC2: (1) snack policy – fresh fruits and vegetables and whole grains are the snack foods of choice (avoid sweets and high-fat foods); (2) beverage policy – serve low-fat (1 %) or non-fat milk only (no whole milk), serve juice only one time/week, water available all day and encouraged as the beverage of choice; (3) Physical Activity Policy – at least 90 min of physical activity every day; and (4) Screen Time Policy – screen time less than 30 min/week. In addition, a registered dietitian nutritionist provided guidelines for menu planning that were (1) consistent with HC2 policy guidelines; (2) consistent with the Dietary Guidelines for Americans^(23)^ and the Child and Adult Care Food Program^(24)^ meal patterns; and (3) cost-neutral.

##### Child curriculum to support policies

The child curriculum lesson plans for instructional needs were consistent with the HC2 policies above and are based on Caring for our Children 3rd Edition^(21)^ standards as well as messaging from the Let’s Move campaign^(22)^. The lesson plans consisted of physical activities and health-oriented messages that could be seamlessly incorporated into everyday activities given they include cognitive, fine motor and self-help instructional components required in pre-school curriculums.

#### Tier 2 (teacher) and tier 3 (parent) role modelling curriculum

The HC2 role modelling curriculum for parents and teachers is based on Project M.O.M.^(25)^, and the principles of the ‘nutritional gatekeeper’ concept developed by the USDA^(24)^. The parent and teacher curriculums consisted of six monthly workshops that were related to core lesson plan principles.

#### Control arm

CCC randomised to the control arm received an attention control consisting of *Safety Sam*, a character who delivers a safety curriculum that was fully developed and implemented in HC2 Phase 1 by our curriculum specialists. Control CCC received the same pre-post measures and incentives as the intervention arm to ensure retention/reduce loss to follow-up.

### Measurements

#### Environment and Policy Assessment and Observation tool

The Environment and Policy Assessment and Observation (EPAO)^(26)^ tool was the primary outcome measure to examine CCC environmental changes in this analysis. EPAO data were collected pre- and post- HC2 implementation in study years 1 and 2 (for a total of three time points, or pre-school year 1 and post-school year 1 and 2) to assess policy change impact in treatment *v.* control centres. The EPAO includes 145 multi-part questions organised into the following 10 sections: morning meal, activities before lunch, lunch, naptime, afternoon snack, activities after lunch, activities in general, equipment/environment/space, food preparation and additional food details. Similar to other studies^(27)^, this analysis focused on the EPAO total nutrition and physical activity scores, which uses a combination of observation and document review data as the primary exposure. Specifically, the nutrition-related sections of the EPAO are used to assess compliance with thirty-eight nutrition best practices, each of which is rated on a scale of 0–3, where higher scores indicate closer compliance. These best practices are then grouped into seven subscales reflecting various aspects of the overall nutrition environment within CCC. Scores on individual best practices are averaged to determine the subscales score; hence, subscale scores range between 0 and 3. Subscale scores are then summed to determine the total score, with scores ranging from 0 to 21, and higher scores indicating better quality nutrition environments. In secondary analyses, we examined each of the seven nutrition subscales within the EPAO, including foods provided (twelve items), beverages provided (five items), feeding environment (seven items), feeding practices (eight items), menus and variety (one item), nutrition education (four items) and nutrition policy (one item). The physical activity total score was calculated in a similar fashion.

To reduce assessment bias and increase the accuracy and objectivity of all environmental measures, the EPAO tool was triple-blinded in that the observer/recorder team members, the CCC teachers/staff/administrators and the statistician did not know CCC treatment arm assignment.

### Statistical analyses

The CCC was the unit of analysis. Thus, change in EPAO nutrition and physical activity scores from baseline to end of school year 1 and 2 by CCC randomisation arm was analysed. The *P*-value for these changes in EPAO score was computed using a random effect model due to the randomised controlled trial study design. Next, a mixed effect linear regression model with observations ID within cluster as the random effect was fitted to assess the relationship between EPAO total nutrition subscore and total physical activity subscore *v.* control from baseline to 2 years follow-up, or over a total of three time points (beginning and end of school year 1 and end of school year 2). Thus, fitting observation within cluster as random effect resulted in controlling the cluster effect in the association between intervention and control over time and EPAO total nutrition subscore and total physical activity subscore. The variable time was analysed as continuous, and the intervention over time as continuous.

#### Joint model

Separate analyses were performed to explore a joint model that included the combined EPAO nutrition and physical activity subscores. Indeed, the use of multilevel analyses with bivariate outcomes has become common in public health research^(28,29)^. Since modelling both the longitudinal and correlation structure for this score were of interest, these trend analyses were modelled for intervention and control groups separately. With the assumptions that the effect of intervention and the trend of both EPAO physical activity and nutrition scores can be explained by two linear mixed models with correlated random effect, we jointly modelled EPAO physical activity and nutrition scores to better understand the effect of the HC2 intervention. We applied this technique to the EPAO physical activity and nutrition scores, because these dependent variables are likely to be highly correlated. This model takes into consideration all components of variability, that is, the within and between variance of each outcome and the between outcomes variability. Conceptually, the model can be thought of as a hierarchical system of regression equations. The analyses followed this design; the participants within CCC were treated as clustered and thus analysed as a random effect. This approach takes into consideration within- and between-cluster variability as well as between- and within-subject variability. Unlike the classical multi-level analysis, where treatment effect is assessed through the interaction between intervention and time, in joint multi-level analyses, the predictors of interest are the average effect – a measure of this joint additive effect, and the time – the trend of this additive effect.

Without loss of generality, we assume that our EPAO physical activity and nutrition scores are nearly normal and correlated. We used superscripts and for the EPAO physical activity and nutrition score, respectively. Superscripts are used to denote the information of the measurement on the individual, since the subjects were followed over time. With the assumption that the EPAO physical activity and nutrition score trend can be explained by two linear mixed models with correlated random effects, a bivariate model for the marker measurements can be presented as a joint model (see Equation 1)^(30)^:(1)
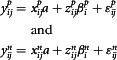

Vectors 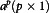 and 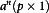 contain fixed regression coefficients for EPAO physical activity and nutrition score, respectively, whereas 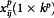 and 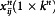 are their corresponding design vectors containing the values of 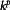 and 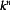 independent variables, respectively. Vectors 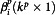 and 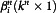 contain random regression coefficients for 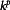 and 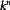 predictor variables included in the corresponding design vectors 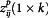 and 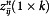 , respectively. The distributions of both 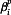 and 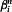 are assumed to be multivariate normal with zero means and variance–covariance matrix:

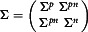

Moreover, 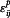 and 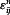 are corresponding level-1 residuals for EPAO physical activity and nutrition score, respectively.

### Covariates of interest

The HC2 intervention *v.* control was the independent variable of interest and was analysed as a binary predictor with two levels, with the control as reference variable. The variable time was analysed as a continuous variable. We included an interaction term for the intervention and control groups over time in the final model.

All statistical analyses were performed in SAS v 9.4 (SAS Institute Inc.).

## Results

As demonstrated in Fig. 1 of the 416 CCC assessed for eligibility, 392 CCC were excluded. Twenty-four CCC were randomised with twelve allocated to the HC2 intervention and twelve allocated to the control (Safety Sam intervention). None of the CCC were lost to follow-up. Accordingly, all CCC were included in the analysis.

Tables 1 and 2 summarise the change in EPAO total nutrition and physical activity subscores over 2 school years and three time points (Fall 2015, Spring 2016 and Spring 2017) by treatment study arm. In general, the treatment CCC showed a greater increase in total nutrition subscore (from 12·36 to 14·43) and physical activity subscore (10·41 to 12·30) *v.* control schools (12·30 to 13·26 and 10·07 to 11·79). The intervention CCC saw the biggest increases in the domains of nutrition education (from 4·20 to 15·89) and nutrition policies (7·35 to 12·48) but also saw a decrease in consumption of fruits and vegetables domain (16·44 to 15·02). Analyses also showed that over the 2 school years, intervention CCC decreased their sedentary environment (13·20 to 9·68) and increased their physical activity environment (9·65 to 12·59), physical activity education (0·98 to 15·17) and physical activity policies (3·76 to 8·51). Interestingly, for many of these domains, the control CCC also had similar changes, but not as well defined as the intervention CCC.

**Table 1:** Change in nutrition EPAO domain score, baseline to 2-year follow-up by randomisation group

	Healthy caregivers, healthy children (HC2) intervention centres (*n* 12)								Safety curriculum attention control centres (*n* 12)							
EPAO domains	Fall 2015		Spring 2016		Spring 2017		Change*		Fall 2015		Spring 2016		Spring 2017		Change*,†	
Nutrition	Mean	sd	Mean	sd	Mean	sd	*P*		Mean	sd	Mean	sd	Mean	sd	*P*	
Fruits and vegetables	16·44	0·13	17·70	0·10	15·02	0·19	**−0·46**	0·06***	16·46	0·13	13·80	0·17	15·33	0·28	**−2·97**	0·07***
Grains	14·38	0·10	12·43	0·17	14·24	0·30	−0·08	0·08	11·90	0·14	12·10	0·20	12·45	0·24	**0·20**	0·07**
High sugar, salt and fat	14·54	0·14	15·37	0·09	14·94	0·10	**0·30**	0·05***	14·46	0·11	12·34	0·14	14·96	0·19	0·19	0·07
Beverages	16·27	0·10	16·05	0·09	16·15	0·23	−0·06	0·05	15·99	0·12	15·01	0·16	15·12	0·23	**−0·27**	0·06***
Nutrition environment	10·99	0·16	10·91	0·17	10·81	0·26	0·01	0·09	10·78	0·20	10·79	0·24	13·15	0·08	**0·72**	0·08***
Nutrition staff behaviours	14·77	0·09	13·75	0·17	15·91	0·27	**0·34**	0·08***	16·12	0·14	15·58	0·25	17·47	0·21	**0·37**	0·07***
Nutrition education	4·20	0·08	13·98	0·22	15·89	0·29	**4·05**	0·12***	2·84	0·19	7·29	0·33	8·82	0·33	**1·86**	0·12***
Nutrition policies	7·35	0·18	8·40	0·32	12·48	0·51	**1·75**	0·10***	9·78	0·31	9·91	0·30	8·74	0·46	**−0·47**	0·13***
Total nutrition subscore	12·36	0·05	13·57	0·07	14·43	0·07	**0·73**	0·03***	12·30	0·08	12·10	0·13	13·26	0·13	**0·29**	0·04***

EPAO, Environment and Policy Assessment and Observation.

* Boldface indicates statistical significance (**P < 0 01, ***P < 0 001) for overall change from Fall 2015 to Spring 2017.

† Estimate of the total change over all three time points over 2 school years.

**Table 2:** Change in physical activity EPAO domain score, baseline to 2-year follow-up by randomisation group

	Healthy caregivers, healthy children (HC2) intervention centres (*n* 12)								Safety curriculum attention control centres (*n* 12)							
EPAO domains	Fall 2015		Spring 2016		Spring 2017		Change*		Fall 2015		Spring 2016		Spring 2017		Change*,†	
Physical activity	Mean	sd	Mean	sd	Mean	sd	*P*		Mean	sd	Mean	sd	Mean	sd	*P*	
Active time	12·24	0·20	7·69	0·18	10·79	0·35	**−0·78**	0·12***	13·28	0·17	8·64	0·25	8·63	0·33	**−1·64**	0·10***
Sedentary time	14·42	0·17	12·60	0·19	15·32	0·45	**0·34**	0·10**	12·83	0·15	10·10	0·20	16·42	0·30	**1·24**	0·11***
Sedentary environment	13·20	0·19	11·14	0·26	9·68	0·54	**−1·00**	0·10***	10·91	0·34	11·48	0·19	12·84	0·52	**0·45**	0·14**
Physical activity environment	10·33	0·20	9·65	0·18	12·59	0·19	**0·74**	0·08***	11·73	0·21	11·89	0·36	12·59	0·34	0·03	0·09
Play equipment	13·60	0·13	11·31	0·16	11·42	0·31	**−0·89**	0·06***	10·38	0·20	13·64	0·21	14·35	0·27	**1·15**	0·08***
Physical activity staff behaviours	14·75	0·30	11·60	0·17	14·89	0·41	−0·23	0·12	15·34	0·21	13·86	0·33	16·50	0·30	0·28	0·11
Physical activity education	0·98	0·10	11·70	0·15	15·17	0·19	**5·01**	0·12***	0·41	0·07	3·36	0·30	5·57	0·27	**1·55**	0·08***
Physical activity policies	3·76	0·29	1·02	0·23	8·51	0·82	**1·14**	0·16***	5·77	0·52	0·0	0·0	7·43	0·80	0·38	0·25
Total physical activity subscore†	10·41	0·09	9·59	0·08	12·30	0·13	**0·54**	0·05***	10·07	0·11	9·12	0·15	11·79	0·23	**0·43**	0·06***

EPAO, Environment and Policy Assessment and Observation.

* Boldface indicates statistical significance (**P < 0 01, ***P < 0 001) for overall change from Fall 2015 to Spring 2017.

† Estimate of the total change over all three time points over 2 school years.

**Fig. 2:**
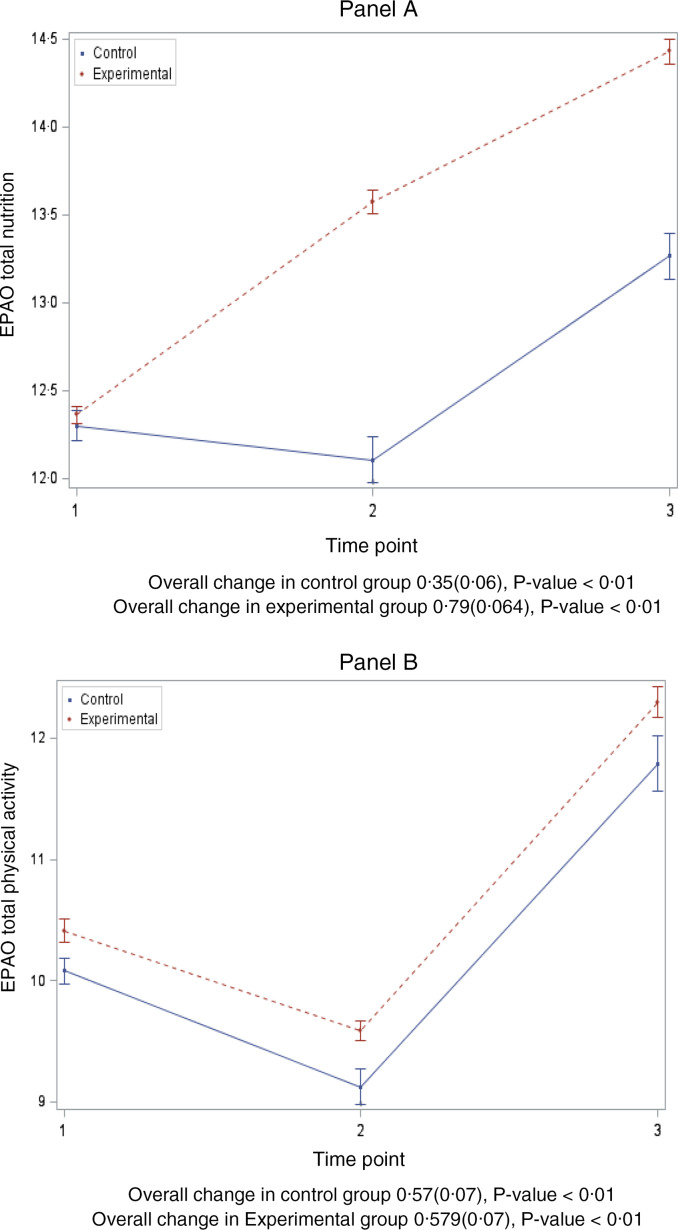
EPAO total nutrition and physical activity score changes over 2 school years by randomisation group, Healthy Caregivers – Healthy Children study (2015–2017)

Figure 2 shows the overall change in EPAO total nutrition score (Panel A) and total physical activity score (Panel B). While both treatment and control CCC improved in both domains, the experimental/HC2 group’s improvement in nutrition environment was almost twice that of the control CCC (0·79 *v.* 0·35) while only slightly improved in physical activity environment *v.* control CCC (0·59 *v.* 0·57) at the end of the 2-year intervention. Regardless, both groups had significant improvements in both domains over the 2-year intervention (*P* < 0·01).

**Fig. 3:**
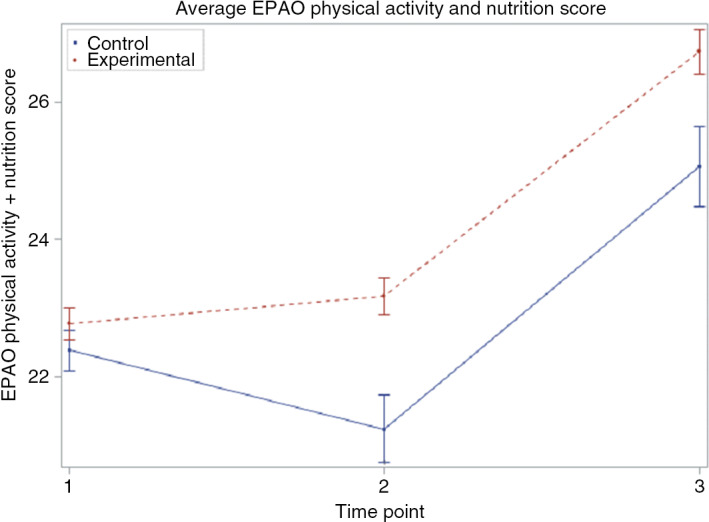
Average EPAO physical activity and nutrition combined scores and the corresponding 95 percent CI

In Table 3, we present results from the bivariate multilevel model for the outcome CCC mean *combined* EPAO physical activity and nutrition subscores over time. The results of the average effect here signal the effect of both the EPAO physical activity and nutrition score over time. Figure 3 is a pictorial presentation of the mean EPAO physical activity and nutrition score and the corresponding 95 % CI. There was a significant effect of CCC intervention on EPAO physical activity and nutrition score as compared to the control. Overall, there was a significant decrease in the joint EPAO physical activity and nutrition over time, that is, from inception to termination of the study. EPAO physical activity and nutrition intake increased with age.

**Table 3:** Bivariate multilevel model of the within and between correlation structures of the EPAO scores together with the longitudinal process

Effect	Parameter estimate	95 % CI	*P*-value
Average effect of (physical + nutrition score)	2·00	1·81, 2·20	<0·001
Time	−0·09	–0·15, –0·03	<0·001
Intervention *v*. Control over time	0·09	0·00, 0·18	0·05

EPAO, Environment and Policy Assessment and Observation.

## Discussion

Results here show that obesity prevention interventions can have a positive impact on the nutrition environment in the CCC setting to promote healthy weight in the early childhood years, and perhaps lower the risk of obesity in this important developmental period, and among high risk groups (e.g. low resource and ethnic minority) in particular. However, these results also show that CCC may need consistent support for improving the physical activity environment over multiple school years to ensure that the environmental and policy changes remain intact to be effective.

The results of this study show that an obesity prevention programme targeting the CCC environment can have an impact on the quality of dietary intake at the centres, and namely an increase in whole-grain consumption. These findings were similar to the results of an umbrella review of healthy eating interventions in childcare settings; in this review, child dietary food intake and food choices were influenced most by interventions that were multi-component, addressed physical activity and diet, targeted individual-level and environmental-level determinants and engaged parents^(14)^. While HC2 emphasises fresh fruit and vegetable intake, analysis showed a decrease over the 2-year intervention. Interestingly, the same trend was seen in the control CCC, suggesting that the access and/or cost of affordable fresh produce and vegetables in particular may have increased over the 2-year study period making it less attractive, or even less realistic for CCC purchase^(31)^. Both control and intervention CCC are located in low-income, food desert/swamp areas with little access to larger grocery chains that provide vendor access to fresh fruits and vegetables on a daily basis. The additional cost of transportation/gas for vendors to purchase fresh produce for the CCC may have been prohibitive for their profit margins, and thus they chose to decrease delivery over time. Also of interest, while not significant, the high sugar, salt and fat domain increased in the intervention CCC from Fall 2015 to Spring 2016 and then fell again in Spring 2017. This may be explained by providers substituting sweet and/or salty snacks (that were also high in fat) for fresh fruits and vegetables when they were too costly. It may have also taken CCC over a year to adjust and settle into the CCC curriculum to see permanent healthy changes in menus. These are important findings given that low-resource children consume the majority of their weekday meals (and meals overall) in the CCC setting, and thus they may not be meeting the USDA daily recommendations for fruit and vegetable intake and consuming more high sweet and salty snacks with high fat content as a result^(12,13)^.

The total nutrition subscore increase in the intervention group was largely driven by nutrition education, which saw the largest gain in subdomain score over the 2-year study period, followed by nutrition policy (comprehensive policy as HC2 included a snack and beverage policy). Similarly, the American Dietetic Association provided nutrition benchmarks for children aged 2 to 5 years attending childcare and suggests that providers should educate children on the origin of food through books, posters, and hands-on experiences and provide exposure to food by engaging their senses^(32)^.

Positive changes in the physical activity environment in CCC treatment schools were largely driven by a significant increase in physical activity education and policies. Interestingly, the survey item on increase in play equipment did not seem to impact the overall increase in physical activity and a decrease in sedentary time. In fact, the control CCC had acquired more play equipment than the treatment CCC over the 2-year study period but saw a decrease in active time (as did the intervention CCC). This could possibly be attributed to the strict licensing codes established for playgrounds rendering them less interesting and challenging for children^(33)^. Once all eight physical activity domains were taken into account, overall curves were parallel for both treatment groups with a decrease from the beginning of year 1 to the end of year 1 and then a steep increase in year 2. This may suggest that CCC need at least one, and preferably two, school years to adjust to new physical activity environment changes. The majority of CCC interventions that have implemented physical activity policies and practices have elicited a centre-level change that unfortunately did not translate to child-level changes^(12)^. However, Stephens *et al.* (2014) found that children who attended New York City CCC that were more adherent and consistent in implementing policies were also more individually active.

We also found that the CCC environment in the HC2 intervention group is associated with an increased EPAO physical activity and nutrition *combined* score over time. The total nutrition and physical activity subscore increases were largely driven by the education subdomains which saw the largest increases in both the intervention and control groups. Given the current obesity epidemic and health-related consequences, it is critical to engage young children in healthy lifestyle habits to prevent chronic disease later in life, and these findings indicate that health education is a useful tool in health promotion within this context. Additionally, the significant change and slight increase found among the nutrition and physical activity education in the intervention and control group through the duration of the study underscore the importance of the role of education in interventions like these. These findings are beneficial to the creation and implementation of obesity prevention efforts in community-based settings.

It should be noted that the control CCC also improved both nutrition and physical activity subscores over the 2-year period. It may have been possible that the control CCC were exposed to other county-wide healthy weight messaging during the project. For example, the Consortium for a Healthier Miami Dade was a county health department effort to promote healthy weight from the early childhood years through adulthood that targeted low-resource communities. This finding may suggest that greater awareness to public health campaigns alone can lead to other healthful improvements, which would be a logical and fascinating extension of this work.

Past research which involved EPAO scores in analysis utilised a multilevel separate modelling approach^(34–36)^. For example, several physical activity subscores of the EPAO scheme have been found to be significantly associated with an increase in moderate to vigorous physical activity levels in children. However, this study utilised correlation coefficient testing rather than regression modelling and measured each physical activity subscore separately^(34)^. These findings have been supported in other studies but have yet to be investigated jointly with nutrition^(35)^. Similarly, other studies have indicated that healthy childhood nutrition practices are positively associated with supportive and constructive provider food practices, demonstrating that the early childcare setting plays a vital role in influencing eating habits in children^(36)^. However, these findings were not analysed in conjunction with physical activity. The results of this study reaffirm that joint modelling is a useful and unique approach for the prediction of correlated habit-forming behaviours in young children.

Our overall findings suggest that the H2 intervention had a combined effect of environmental changes in nutrition and physical activity. However, CCC may need consistent support over time, and over more than 1 school year to ensure that the environmental and policy changes become institutionalised to support healthy weight development. One recent review citing characteristics of effective healthy eating interventions in the CCC environment reported that positive outcomes were ‘mostly facilitated by researchers/external experts’ pointing to the challenge of the permanent adoption of such programmes by CCC^(14)^. Combined with our findings, this conclusion points to the need for more rigorous dissemination and implementation studies in this setting.

### Limitations

The results reported here should be considered in light of some limitations. First, given the span of the study over 2 school years with children changing CCC, matriculating to kindergarten, or no longer being financially eligible to attend, attrition was a challenge. Second, because HC2 targeted low-income children and families, results cannot be generalised to all pre-school children regardless of socio-economic background. Third, this analysis does not account for outside intervention time that may have been attributed to healthy (or unhealthy) behaviours. However, given that all participants were from low-resource backgrounds and many of the children spend the majority of their waking hours and consume the majority of their meals during the week in the CCC, some may argue that the CCC environment has the potential to be equally, if not more influential in establishing healthy lifestyle habits during these critical developmental years.

## Conclusions

Results here show that obesity prevention interventions can have a positive impact on the CCC nutrition and physical activity environment, which in turn can promote healthy weight development in the early childhood years. However, our results also show that CCC may need consistent support over time and multiple school years to ensure that environmental and policy changes are fully integrated to be effective.
